# Contributing to agriculture by using soybean seed data from the tetrazolium test

**DOI:** 10.1016/j.dib.2018.12.090

**Published:** 2019-01-04

**Authors:** Douglas F. Pereira, Pedro H. Bugatti, Fabricio M. Lopes, André L.S.M. Souza, Priscila T.M. Saito

**Affiliations:** a Department of Computing, Federal University of Technology - Paraná, Parana, Brazil; b Belagricola Enterprise, Parana, Brazil; c Institute of Computing, University of Campinas, Sao Paulo, Brazil

**Keywords:** quality control, soybean seed data, tetrazolium test, classication, visual features

## Abstract

Agribusiness has a great relevance in the world׳s economy. It generates a considerable impact in the gross national product of several nations. Hence, it is the major driver of many national economies. Nowadays, from each new planting to harvesting process it is mandatory and crucial to apply some kind of technology to optimize a given singular process, or even the entire cropping chain. For instance, digital image analysis joined with machine learning methods can be applied to obtain and guarantee a higher quality of the harvest, leading to not only a greater profit for producers, but also better products with lower cost to the final consumers. Thus, to provide this possibility this work describes a visual feature dataset from soybean seed images obtained from the tetrazolium test. This is a test capable to define how healthy a given seed is (e.g. how much the plant will produce, or if it is resistant to inclement weather, among others). To answer these questions we proposed this dataset which is the cornerstone to provide an effective classification of the soybean seed vigor (i.e. an extremely tiresome human visual inspection process). Besides, as one of the most prominent international commodity, the soybean production must follow rigid quality control process to be part of world trade. Hence, small mistakes in the seed vigor definition of a given seed lot can lead to huge losses.

## Specifications table

**Table:** 

Subject area	Computer Science, Agronomy, Soybean crop
More specific subject area	Image Analysis, Soybean Seeds, Tetrazolium Test
Type of data	Image features (numerical data)
How data were acquired	Visual feature extraction from the seed images obtained through the tetrazolium test.
Data format	Floating point n-dimensional vectors for each image
Experimental factors	Description and classification of the soybean seed damages.
Experimental features	In the tetrazolium test, the seeds are cut in half and the 4 parts of the seed are analyzed (2 internal portions and 2 external portions). These parts were scanned, generating seed sheets that comprise several seed images. Each image was annotated by a seed analyst. 1,758 images were captured in two sessions in a company׳s seed analysis laboratory.
Data source location	The seeds were scanned and annotated in the seed analysis laboratory in Tamarana, Paraná, Brazil. The preprocessing and feature extraction phases occurred at the Federal University of Technology - Paraná, in Cornélio Procópio, Paraná, Brazil.
Data accessibility	Data is publicly available on github (https://github.com/BioinfoCP/visual-features-soybean-vigor).
Related Research Article	Pereira et al. [1]. An image analysis framework for effective classification of seed damages. Proceedings of the 31st Annual ACM Symposium on Applied Computing (SAC), ACM, 2016, pp. 61–66.

## Value of the data

• The first open-access visual feature dataset that describes characteristics of soybean seeds obtained from the tetrazolium test;
• Our dataset provides different types of color and texture-based visual features to the research community. Thus, it is possible to analyze which type of feature is better according to each seed damage, its level and seed portion;
• Our visual features allow the automatic classification of seed damages, enhancing and aiding in a great extent the work performed by the seed analysts;
• The dataset enables an effective way to the automatic definition of the soybean seed vigor through machine learning and data mining methods fine-tuned to the seed vigor context;
• Useful not only for different researchers, but also for farmers around the world to obtain a simple and efficient decision aided process regarding the seeds׳ quality that they buy from the seed producers. Besides, it allows the definition of counter-proof systems, aiding the seed analyst and the farmer against possible mistakes or deliberate alterations aiming for profit.

## 1 Data

This dataset contains information and visual features of the images of soybean seeds classified according to the damages and the intensities of the damage obtained from the tetrazolium test [2], [3]. The damage classes considered were mechanical, bug, humidity and no damage (i.e. perfect seed) and damage intensities up to level 3.

**Fig. 1:**
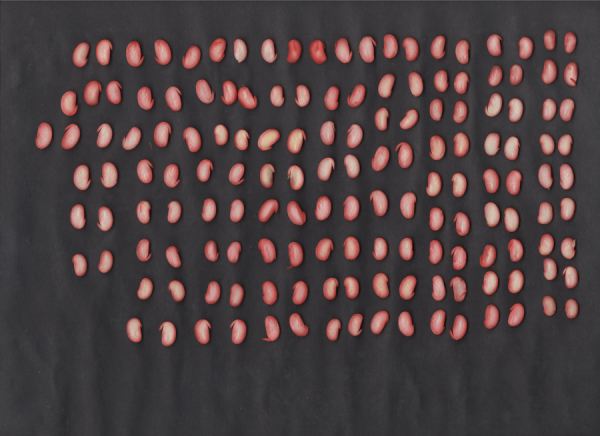
Example of a sheet with seeds.

**Fig. 2:**
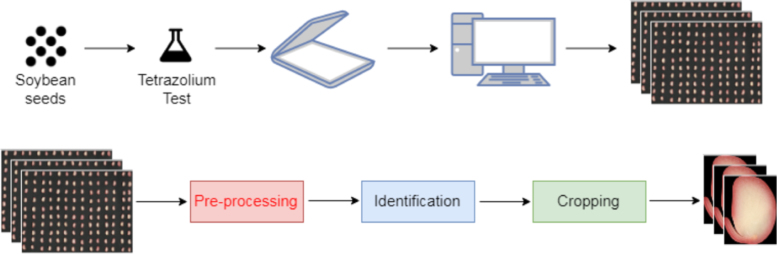
Pipeline adopted for processing the seed sheets.

**Fig. 3:**
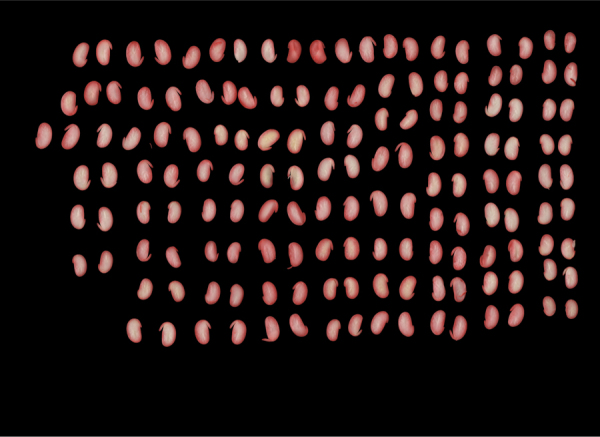
Seed sheet after preprocessing.

**Fig. 4:**
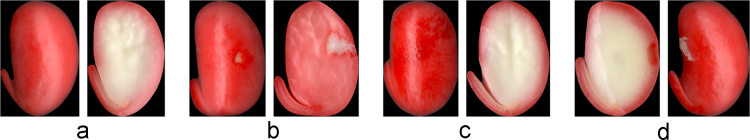
Examples of external and internal portions of seed samples. (a) without damage (perfect). (b) with bug damage. (c) with humidity damage. (d) with mechanical damage.

The acquisition of the seed images generated sheets (Fig. 1). The images undergo a processing pipeline (Fig. 2) in order to remove noise and improve them (Fig. 3). After a cropping process, we obtained individual images of soybean seeds (Fig. 4) separated by classes of damage.

Color-based and texture-based visual features were extracted from each seed image. To obtain the color-based features, we employed the border-interior classification (BIC) [4], and the global color histogram (GCH) [5]. The color-based extraction were performed using the RBG color space. The Haralick [6] aggregated with the co-occurrence matrix and the local-binary pattern (LBP) [7] descriptors were applied to obtain the texture-based features. Table 1 details the description of each feature extractor, their respective types and number of features.

**Table 1:** Description of the extractors, types and number of features.

Extractor	Description	Type	#Features
BIC [4]	Border/interior classification	Color	128
GCH [5]	Global color histogram	Color	66
Haralick [6]	Haralick׳s descriptors	Texture	5
LBP [7]	Local binary patterns	Texture	256

Finally, Tables 2 and 3 present the description and distribution of samples of each image class obtained.

**Table 2:** Description and distribution of samples of each image class obtained in the first acquisition.

Classes	Description	Samples
0PE	External portion w/o damage (perfect)	502
0PI	Internal portion w/o damage (perfect)	529
2HE	External portion w/ humidity damage - level 2	23
2HI	Internal portion w/ humidity damage - level 2	7
3ME	External portion w/ mechanical damage - level 3	36
3MI	Internal portion w/ mechanical damage - level 3	28
3BE	External portion w/ bug damage - level 3	83
3BI	Internal portion w/ bug damage - level 3	40
3HE	External portion w/ humidity damage - level 3	36
3HI	Internal portion w/ humidity damage - level 3	49

**Table 3:** Description and distribution of samples of each image class obtained in the second acquisition.

Classes	Description	Samples
0PE	External portion w/o damage (perfect)	306
0PI	Internal portion w/o damage (perfect)	374
3ME	External portion w/ mechanical damage - level 3	4
3MI	Internal portion w/ mechanical damage - level 3	5
3BE	External portion w/ bug damage - level 3	18
3BI	Internal portion w/ bug damage - level 3	17
3HI	Internal portion w/ humidity damage - level 3	9

## 2 Experimental design, materials, and methods

### 2.1 Tetrazolium test

Agriculture in recent years has been gathering efforts to find solutions that enable the increase of productivity of cultivars. The seeds are the basic and necessary inputs for agricultural production. The germination test is one of the tests applied to evaluate the quality of the seed, but it does not provide information about the vigor and/or longevity of the seed.

The seed vigor answers how healthy and vigorous the seed is. Hence, based on this test it is possible to obtain how much the plant will produce according to varying type of pests and climate conditions.

Considering soybeans, the test basically consists of preconditioning the soybean seeds in a germinating paper where they are moistened for 16 h at a temperature of 25°C or for 6 h at a temperature of 41°C. After this preconditioning, the seeds are immersed in a solution called tetrazolium salt in the concentration of 0.075% for approximately 150 to 180 min to acquire the coloration that shows the damages in the seed. Then, analysts need to cut the seeds of a given lot in half and analyze them one by one to define their possible damages. A sampling is applied to each lot, leading to 200 seeds that represent the entire lot. It is estimated that each analyst performs the analysis of 30 to 40 lots per day.

### 2.2 Acquisition and preprocessing of images

Two sessions were carried out at the seed analysis laboratory at the company׳s Belagrícola in Tamarana, Parana unit, where analysts carried out the tetrazolium test on some seeds to perform the image acquisition.

The images were acquired using an EPSON L355 all-in-one scanner at 1200dpi resolution. The seeds were placed in a matrix with the black background and submitted to the scanning. Fig. 1 shows an example of a scanned sheet with soybean seeds.

For processing of the images (Fig. 2), three procedures were applied, including preprocessing, identification and cropping [1].

The preprocessing procedure aims to remove the irregular background of the images, as well as the noise generated from the acquisition process. Since the seeds, that pass through the tetrazolium test process, have a characteristic of reddish color, initially, the image undergoes a transformation in the color space of RGB (Red, Green, Blue) for HSV (Hue, Saturation, Value). The H-channel (hue) allows a better representation and manipulation of the colors of the images.

Next, the image undergoes a segmentation process based on threshold of the H channel in the intervals of 0° to 60° and of 300° to 360°, that correspond to the intensities of colors between the yellow and the magenta. After this threshold-based segmentation, morphological operations are applied to the original image to obtain the image only with the seeds (i.e. without the irregular background), as illustrated in Fig. 3.

For the procedure of identifying the seeds on the sheet, a contour detection operation is performed. For each set of pixels found in the image, an ellipse that fits into that set of pixels is calculated. This ellipse is created considering a conic detection method based on algebraic distance [8]. Afterwards, it is created an internal bounding box that wraps the detected ellipse. Then, a second bounding box (named here as external) is generated, enveloping the first one. This new bounding box has its *y*-axis aligned at 90° and *x*-axis at 0°.

The external bounding box is used to crop the seed image. In some cases, the external bounding box does not guarantee that the seed image is fully contained in it. Therefore, this bounding box is increased by 50% of its dimensions. Then, a cropping operation is applied to the image with the dimensions of the external bounding box increased. Thus, as a result, it is obtained a set of individual seed images.

### 2.3 Dataset description

Fig. 4 shows examples of external and internal portions of seed images from each class. We obtained 1333 and 733 images (referring to the 4 portions of the seed) in the first and second acquisitions, respectively. Tables 2 and 3 present the descriptions of the classes and the distribution of samples from each image class obtained in the first and second acquisitions, respectively. Damage intensities up to level 3 are considered, due to the obtaining of sufficient quantity of samples for such levels of intensity.

Considering both acquisitions, color-based and texture-based visual features were extracted from each seed image. It is worth to mention that with our visual feature dataset it is possible to create different datasets. For instance, it is possible to build datasets considering not only just the first acquisition or the second one, but also a merging between them.

In order to provide an extensive experimental evaluation and consequently improvements in the quality control process, we also presented different ways to explore the dataset, considering different settings.

Other spin-off datasets (subsets) can be also generated considering different classes under analysis. Since the images are classified into a type of damage, its respective level and portion, it is possible to generate datasets isolating one type of damage and consider its different levels. For example, a dataset composed of bug damages with different levels (e.g. 2 to 3). The same process can be applied to the damage level and to the portion. Thus, different kinds of machine learning process can be created according to the demand of the user, and a given context.

## Appendix A Supporting Information

Application 1
